# A paternal lactate dehydrogenase critically enhances male gametogenesis and malaria transmission

**DOI:** 10.1038/s41598-025-05832-1

**Published:** 2025-07-02

**Authors:** Annie Z. Tremp, Sadia Saeed, Johannes T. Dessens

**Affiliations:** Department of Infection Biology, Faculty of Infectious and Tropical Diseases, London School of Hygiene and Tropical Medicine, Keppel Street, London, WC1E 7HT UK

**Keywords:** Plasmodium, Gametogenesis, Glycolysis, Lactate dehydrogenase, Transmission-blocking drugs, Parasite biology, Oxidoreductases

## Abstract

Malaria blood stage parasite development relies on glycolysis to generate ATP, which requires pyruvate to lactate conversion by an essential lactate dehydrogenase enzyme (LDH1). Conversely, parasites developing in the mosquito employ mitochondrial chemiosmosis for ATP production. The source of ATP during transition from vertebrate to insect is less clear; gametes form in the mosquito midgut lumen within minutes of gametocyte ingestion, and while female gametes possess a mitochondrion, this organelle is absent from male gametes (microgametes). Here, we investigate a second LDH enzyme (LDH2) found exclusively in male gametocytes and microgametes. Knockout of *Plasmodium berghei* LDH2 expression reduces the number and size of exflagellation centres and radically diminishes oocyst development in *Anopheles stephensi* mosquitoes. Our data indicate that LDH2 supplements LDH1 activity to facilitate the cytokinesis step of male gametogenesis, while LDH1 alone is sufficient for motility of free-swimming microgametes. Our results point to a key role for glycolytic ATP production in microgamete formation and function and identify LDH activity as a potential malaria transmission-blocking drug target.

**Supplementary Information:**

The online version contains supplementary material available at 10.1038/s41598-025-05832-1.

## Introduction

Intraerythrocytic malaria parasites use glycolysis instead of mitochondrial respiration for their ATP production^1–3^. Glycolysis converts glucose into pyruvate, which requires reduction of the cofactor NAD^+^ to NADH. When NAD^+^ concentration becomes rate-limiting, pyruvate is converted into lactate by an essential blood stage-expressed lactate dehydrogenase (LDH)^4,5^ which oxidises NADH to NAD^+^ allowing glycolysis and resulting ATP production to continue. LDH-catalysed reactions are reversible and therefore lactate is transported out of the cell, a process that can contribute to hyperlactatemia and lactic acidosis in patients with severe malaria^6^.

Malaria parasite transmission starts when *Anopheles* female mosquitoes ingest gametocytes during blood feeding on an infected host. Within seconds, gametocytes are activated and begin a rapid process of gamete formation followed by fertilization in the mosquito midgut lumen. Within a day, zygotes transform into motile ookinetes that traverse the midgut epithelium and transform into oocysts. Over the next two-week period, oocysts undergo growth and division by a process called sporogony to produce thousands of daughter cells named sporozoites. After egress from the oocysts, sporozoites migrate to and colonise the salivary glands of the insect, allowing them to enter the vertebrate host by mosquito bite to initiate new malaria infections.

The mosquito-resident stages of the parasite switch from glycolysis to the more efficient mitochondrial chemiosmosis for their ATP production. In preparation for this, the gametocytes’ mitochondria enlarge, develop cristae and activate the citric acid cycle to feed oxidative phosphorylation^1,7–9^. Consistent with these observations, mutant parasites that are defective in their electron-transport chain or that possess defective mitochondrial ATP synthase display discernible phenotypes only in the mosquito and cannot complete transmission^2,10,11^.

During gametogenesis, female gametocytes each produce a single female gamete, whereas male gametocytes re-enter the cell cycle and replicate their genome in preparation for three rounds of endomitosis^12^. Concomitant with the latter is the assembly of eight axonemes, culminating in the formation of eight sperm-like, flagellated male gametes (microgametes) from each male gametocyte parent. The final cytokinesis step of male gametogenesis, called exflagellation, involves intense flagellar beating to facilitate the budding and subsequent release of the microgametes from the parental plasma membrane. Following exflagellation, microgametes use free swimming to seek out and fertilise female gametes, processes that involve further flagellar activity^13–15^.

To drive flagellar activity, significant ATP production is needed^16,17^ but contrary to female gametes, microgametes do not possess a mitochondrion^7,18,19^. Furthermore, knockout of mitochondrial ATP synthase activity in *P. berghei* does not affect microgamete formation or fertility^2^ indicating that mitochondrial ATP production is dispensable for these processes. Thus, like the asexual blood stages, microgametes are likely to use glycolysis combined with homolactic fermentation for their ATP production. However, direct evidence for this has been lacking, partly because of the essential nature of blood stage parasite-expressed LDH and its refractoriness to gene disruption. In this study we describe a second LDH enzyme solely expressed in male gametocytes and show that it has a vital role in male gametogenesis and parasite transmission.

## Results and discussion

### *Plasmodium* encodes a second LDH

**Fig. 1:**
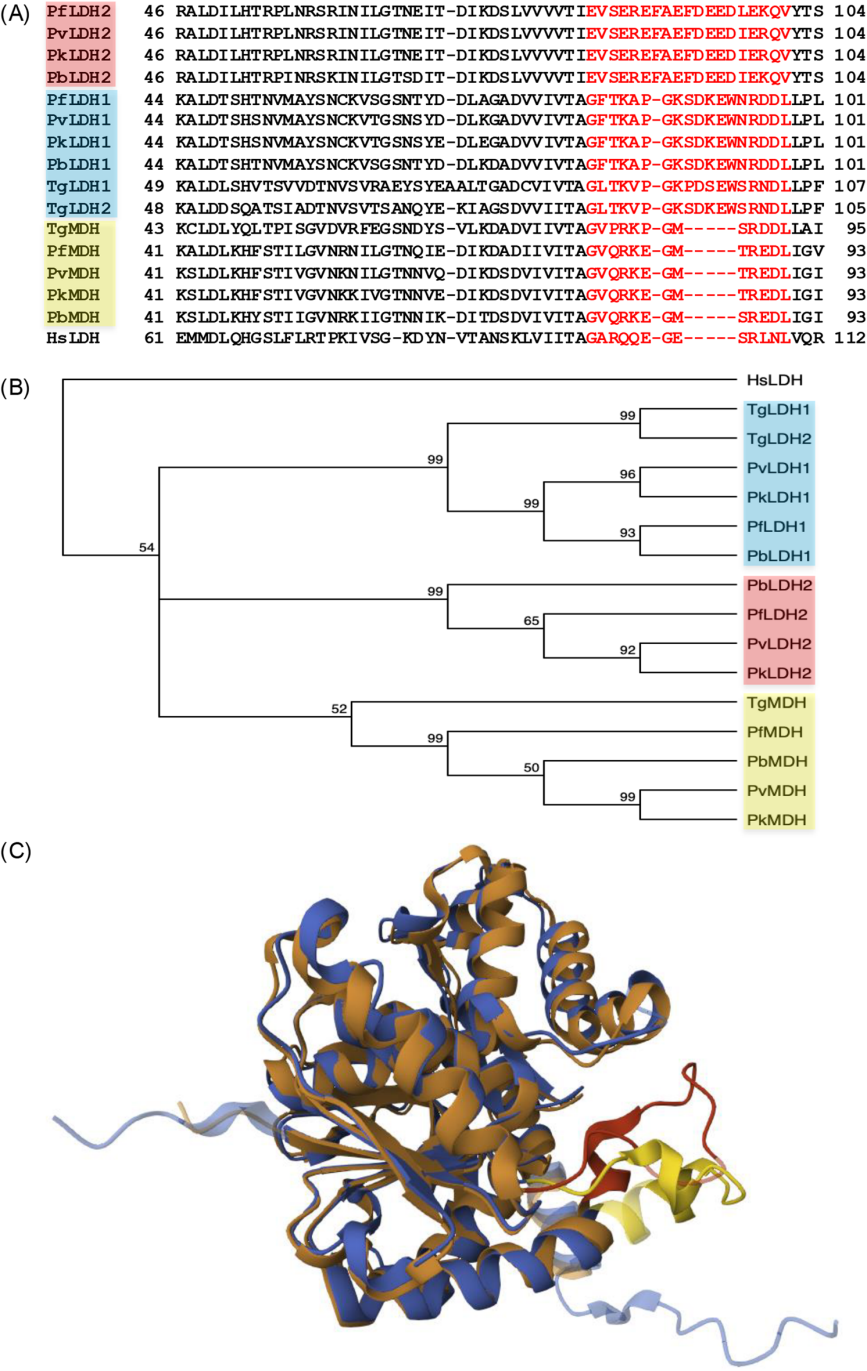
*Plasmodium* encodes a second lactate dehydrogenase (LDH). (**A**) Part multiple sequence alignment of LDH and malate dehydrogenase (MDH) from *Plasmodium*, *Toxoplasma* and human, spanning the substrate binding loops (red). PfLDH1, *P. falciparum* LDH1 (PF3D7_1324900); PvLDH1, *P. vivax* LDH1 (PVP01_1229700); PkLDH1, *P. knowlesi* LDH1 (PKNH_1203900); PbLDH1, *P. berghei* LDH1 (PBANKA_1340100); TgLDH1, *T. gondii* LDH1 (TGME49_232350); TgLDH2, *T. gondii* LDH2 (TGME49_291040); PfLDH2, *P. falciparum* LDH2 (PF3D7_1325200); PvLDH2, *P. vivax* LDH2 (PVP01_1229400); PkLDH2, *P. knowlesi* LDH2 (PKNH_1203600); PbLDH2, *P. berghei* LDH2 (PBANKA_1340400); PfMDH, *P. falciparum* MDH (PF3D7_0618500); PvMDH, *P. vivax* MDH (PVPO1_1131000); PkMDH, *P. knowlesi* MDH (PKNH_1131900); PbMDH, *P. berghei* MDH (PBANKA_1117700); TgMDH, *T. gondii* MDH (TGME49_318430); HsLDH, *Homo sapiens* lactate dehydrogenase A (KAI4070372). (**B**) Phylogeny of the sequences shown in (**A**). Sequences with LDH1-type (blue), LDH2-type (red) and MDH-type (yellow) substrate binding loops are highlighted. Bootstrap values (*n* = 1000) are indicated at nodes. Rooted on HsLDH as outgroup. (**C**) Pairwise structure alignment of *P. falciparum* LDH1 (PDB: 1T2D, maroon) and the AlphaFold-predicted structure of *P. falciparum* LDH2 (Q8IE66, blue). Amino acid identity is 43% across 302 aligned residues, root mean square deviation is 1.03. Substrate binding loops are highlighted in red (LDH1) and yellow (LDH2).

BLAST similarity searches of the *P. berghei* genome with blood stage parasite-expressed LDH (PBANKA_1340100, here named LDH1) revealed the existence of a second LDH (PBANKA_1340400, here named LDH2) that displays high levels of sequence homology as well as orthologous conservation and synteny across *Plasmodium* species (Fig. 1A, Supplementary Fig. S1). *P. berghei* LDH2 constitutes a 334 amino acid protein encoded by a three-exon gene located only three gene positions downstream of LDH1, pointing to a relatively recent gene duplication event having given rise to these paralogues. The canonical active site residues Arg171, His195, Ser245 are conserved between LDH1, LDH2 and closely related malate dehydrogenase (MDH) enzymes, but active site residue Asp168 is substituted for a histidine residue in all LDH2 molecules (Supplementary Fig. S1). Sequence alignment also revealed an extended substrate specificity loop in LDH2 that is absent from mammalian LDHs and from all MDHs (Fig. 1A)^20^. An extension of the substrate binding loop is also found in *Plasmodium* LDH1 and in other apicomplexan LDH proteins like those of *Toxoplasma* (Fig. 1A)^5,20,21^. Phylogenetic analysis of the sequences aligned in Fig. 1A split them according to the three types of substrate binding loops revealed by multiple sequence alignment: LDH2-specific, LDH1-specific, and MDH-specific (Fig. 1B) (the second LDH of *T. gondii*, TgLDH2, is of the LDH1-type). Pairwise structure alignment of the AlphaFold-predicted 3D structure of *P. falciparum* LDH2 (AlphaFold: Q8IE66) with the experimentally determined 3D structure of LDH1 (PDB: 1T2D)^5^ confirmed the high level of structural similarity between these two molecules (Fig. 1C) and the same was true for pairwise structure alignment of LDH1 and LDH2 orthologues in *P. berghei* (Supplementary Fig. S2).

### LDH2 is present in male gametocytes and microgametes

**Fig. 2:**
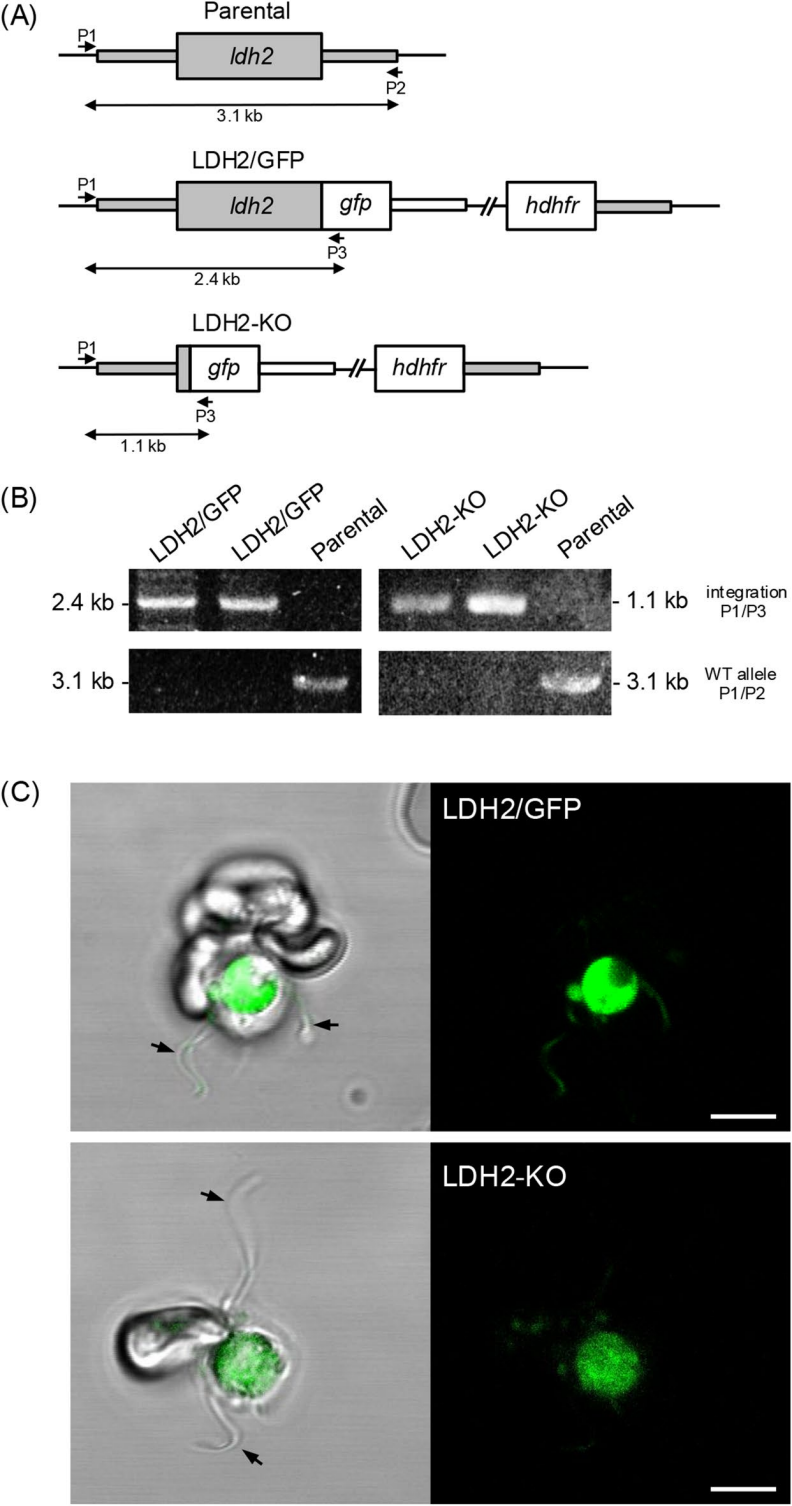
LDH2 is expressed in male gametocytes and microgametes. (**A**) Schematic diagram of the *ldh2* alleles in parental (wildtype) parasites and in the transgenic lines LDH2/GFP and LDH2-KO. The *ldh2* gene is shown in grey with coding sequence (wide bars) and 5′ and 3′ untranslated regions (narrow bars). Also shown are the *gfp* module, the selectable marker (*hdhfr*), and positions of primers P1-P3 used for diagnostic PCR amplification. (**B**) PCR with primers P1 and P3 diagnostic for integration of the modified *ldh2* alleles into the *ldh2* locus, or with primers P1 and P2 diagnostic for absence of the unmodified parental *ldh2* allele. See Materials and Methods section for primer sequences. Corresponding uncropped DNA agarose gels are shown in Supplementary Fig. S3. (**C**) Live fluorescence confocal images of exflagellating LDH2/GFP and LDH2-KO microgametocytes with emerging microgametes (arrows). Scale bar 5 μm.

Transcriptome analysis revealed that *ldh2* transcripts are predominantly present in male gametocytes^22^ pointing to male-specific LDH2 expression. To assess LDH2 protein expression, we used allelic replacement to generate a transgenic *P. berghei* line expressing LDH2 fused at its carboxy terminus to green fluorescent protein (GFP) (Fig. 2A). Diagnostic PCR confirmed integration of the modified *ldh2::gfp* allele into the target locus of the resulting LDH2/GFP parasite line, as well as absence of the parental *ldh2* allele in clonal populations (Fig. 2B, Supplementary Fig. S3). Assessment of GFP expression by confocal fluorescence microscopy of live LDH2/GFP parasites revealed strong fluorescence in exflagellating gametocytes as well as in their emerging microgametes (Fig. 2C), corroborating the male sex-specific transcription pattern of the *ldh2* gene. By contrast, no discernible GFP fluorescence was observed in asexual blood stages, female gametocytes, ookinetes, oocysts and sporozoites (data not shown), confirming that LDH2 is predominantly present in microgametes and their precursor gametocytes. Very recently, male-specific protein expression of LDH2 was also shown for *P. falciparum*^23^.

### LDH2 is important for ookinete and oocyst formation

To study the contribution of LDH2 to parasite development, infectivity and transmission, we generated a null mutant parasite line by allelic replacement, in the process removing the coding sequence of LDH2 except for its amino-terminal 15 residues fused to GFP under control of the native *ldh2* promoter (Fig. 2A). Diagnostic PCR confirmed integration of the *gfp* sequence into the target *ldh2* locus of the resulting LDH2-KO parasite line, as well as absence of the parental *ldh2* allele in clonal populations (Fig. 2B, Supplementary Fig. S3). Correct genetic modification of the LDH2-KO line was further supported by the observation that, as expected, GFP fluorescence was observed in male gametocytes albeit at a lower level than in LDH2/GFP parasites (Fig. 2C). The latter could reflect distinct stabilities of the different-length GFP fusion proteins expressed in these lines (Fig. 2A).

**Fig. 3:**
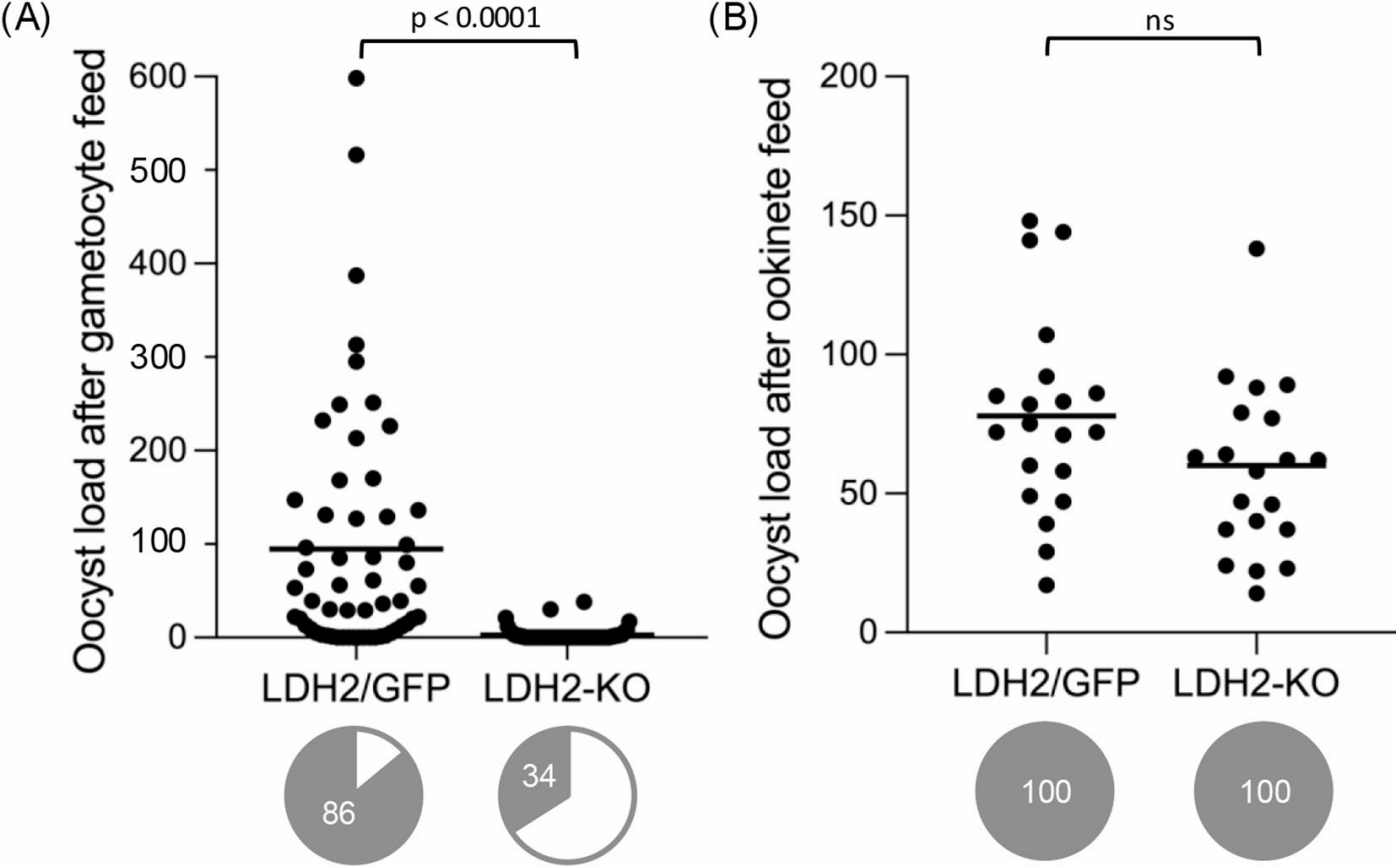
LDH2 promotes oocyst development in *Anopheles stephensi*. (**A**) Oocyst loads in mosquitoes infected with parasite lines LDH2/GFP and LDH2-KO via natural gametocyte feeds. Each scatter plot represents pooled mosquitoes from three independent experiments (*n* = 60). Horizontal lines mark mean oocyst numbers. Pie charts show percentage of mosquitoes with at least one oocyst (prevalence of infection). Only blood-fed mosquitoes were included. Statistical significance (p values, Mann-Whitney) is indicated (ns, not significant). (**B**) Oocyst loads in mosquitoes infected with LDH2/GFP and LDH2-KO via direct ookinete feeds (*n* = 20).

LDH2-KO parasites displayed normal development in the mouse, consistent with the absence of LDH2 expression in asexual blood stage parasites, and they produced male and female gametocytes at similar levels and ratios to their LDH2/GFP counterparts (data not shown). Male gametocytes generated microgametes (Fig. 2C) that displayed in vitro motility with fast flagellar beating indistinguishable from their LDH2/GFP counterparts. Ookinetes were produced in vitro, demonstrating that LDH2-KO microgametes are capable of reaching and fertilising female gametes. Nonetheless, in controlled experiments *Anopheles stephensi* mosquitoes infected with LDH2-KO parasites by natural feeding (on gametocytemic mice) consistently produced markedly reduced (> 30-fold) oocyst numbers compared to insects infected with their LDH2/GFP counterparts (Fig. 3A). If this reduction were reproduced in natural settings, where oocyst numbers are typically very low^24^ LDH2 depletion would likely severely reduce, if not abolish, sporozoite transmission. The dramatic lift in transmission success through the expression of a second, male-targeted LDH activity, would thus have provided a strong selective force for the gene duplication that gave rise to LDH2.

LDH2-KO oocysts displayed normal sporulation and produced sporozoites that could be successfully transmitted by mosquito bite, indicating that LDH2 does not contribute to parasite development downstream of oocyst formation and consistent with the observed absence of LDH2 expression in oocysts and sporozoites. This shows that LDH2-KO parasites can complete the life cycle, at least under laboratory conditions. Proteome studies have shown that *P. berghei* microgametes contain both LDH1 and LDH2 among their most abundant proteins^15^ indicating that LDH1 carried by LDH2-KO male gametocytes and microgametes provides sufficient LDH activity on its own to facilitate microgamete formation, free swimming and fertilisation.

Even though ookinetes were produced in vitro, we reproducibly observed reductions in the number of cultured LDH2-KO ookinetes compared to LDH2/GFP lines (∼3-fold), indicating that LDH2 depletion adversely affected in vitro ookinete formation. However, these reductions could not account for the at least 10 times higher drop in oocyst number in mosquitoes naturally infected with the LDH2-KO parasites (Fig. 3A). To investigate whether this fall in oocyst numbers in LDH2-KO parasite-infected mosquitoes was due to a reduction in ookinete fitness, we infected *A. stephensi* directly with cultured ookinetes via membrane feeders. When comparable ookinete numbers were fed, oocyst numbers obtained were not statistically different between LDH2/GFP and LDH2-KO parasites (Fig. 3B), demonstrating that the *ldh2* gene disruption does not significantly affect ookinete infectivity. This fits with the absence of LDH2 expression in this life stage. Therefore, the large reduction in LDH2-KO oocyst formation in vivo (Fig. 3A) originated from a defect upstream of ookinete development.

### LDH2 promotes male gametogenesis

**Fig. 4:**
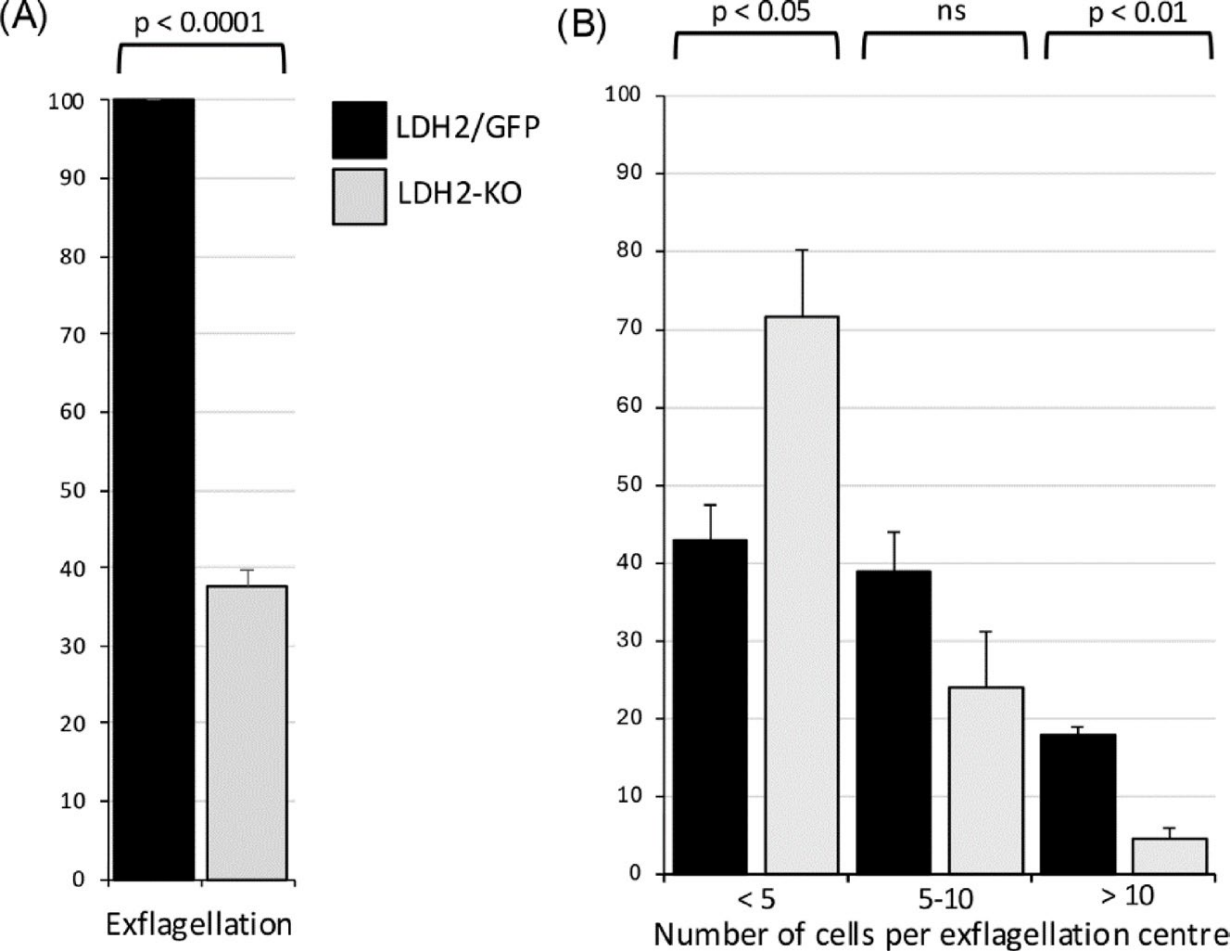
LDH2 promotes male gametogenesis. (**A**) Relative exflagellation levels of LDH2/GFP and LDH2-KO parasite lines (normalised for male gametocytemia, LDH2/GFP exflagellation set at 100%). Each bar represents mean ± sem of three independent experiments. (**B**) Proportion of different size exflagellation centres in LDH2/GFP and LDH2-KO parasites. Each bar represents mean ± sem of three independent experiments, each scoring at least 100 exflagellation events. Statistical significance (*p* values, unpaired *t*-test) is indicated (ns, not significant).

LDH2-KO parasites showed a 3.3-fold reduction in in vitro macrogamete to ookinete conversion compared to their LDH2/GFP counterparts (LDH2/GFP: 62%; LDH2-KO: 19%), consistent with their reduced ookinete formation in culture. Examination of nuclei using Hoechst DNA staining showed that the remaining spherical P28-positive cells were mostly macrogametes in both LDH2/GFP and LDH2-KO populations (data not shown), pointing to a reduction in fertilization events in the latter. Given the male-specific expression of LDH2 (Fig. 2C), the reduced infectivity of the LDH2-KO parasites to mosquitoes could involve a defect in the process of male gametogenesis. Examination of wildtype and LDH2-depleted gametocytes by immunofluorescence imaging, using anti-tubulin antibodies to visualize the flagella, did not reveal discernible differences between the parasite lines (Supplementary Fig. S4), likely reflecting the fact that both are capable of male gametogenesis (Fig. 2C). However, when carefully conducting quantitative assays on gametocyte populations we could observe an LDH2 loss-of-function phenotype. First, examination of exflagellation in vitro consistently revealed significant reductions in overall exflagellation levels of LDH2-KO parasites (∼2.7-fold) when corrected for male gametocytemia (Fig. 4A). Second, we found that male gametogenesis of LDH2-KO parasites produced a significantly higher proportion of small exflagellation centres, as well as a significantly lower share of large exflagellation centres, compared to their LDH2/GFP equivalents (Fig. 4B). Exflagellation centres are formed by the budding microgametes binding to and drawing in neighbouring cells^25^. The presence of both fewer and smaller exflagellation centres in the LDH2-KO parasite indicates that the process of exflagellation is less efficient and results in fewer microgametes completing cytokinesis. Reductions in in vitro exflagellation in LDH2-KO parasites were comparable to the observed reductions in in vitro ookinete development, indicating that LDH2 plays no discernible role beyond exflagellation, for example in male-female gamete fusion, in which case greater reductions in ookinete development in vitro would be expected. Collectively, these observations support a scenario in which LDH2 activity serves to supplement LDH1 activity to boost ATP production specifically for the high-energy cytokinesis step of male gametogenesis.

Successful fertilisation requires encounters between male and female gametes, which microgametes achieve through free swimming to seek out a mate. The adverse effects of LDH2 knockout on male gametogenesis lowers the male to female gamete ratio, which would make encounters between them less likely and result in reduced fertilisation and ookinete numbers. In addition, fertilisation is adversely affected by immune factors in the infected blood meal that attack the emerging gametes, such as macrophages and complement^26^. It is thought that the formation of exflagellation centres promotes fertilisation by creating protective microenvironments for male and female gametes to meet^25^. Accordingly, the reduced size of exflagellation centres in LDH2 knockout parasites could contribute to the lower success of fertilisation, ookinete production and, ultimately, transmission. It is noteworthy that the effect of LDH2 knockout on ookinete development is considerably greater in vivo than in vitro (∼10-fold), indication that it is easier for microgametes to achieve fertilisation in culture. One explanation is that microgametes can move around to find a mate more easily in culture conditions, where the blood is diluted, as opposed to the blood bolus in the *Anopheline* midgut lumen where the cells become concentrated and are tightly packed. Furthermore, immune and other adverse factors present in the infected blood are concentrated in the mosquito blood meal, which could cause greater gamete loss in the insect. Accordingly, a reduced male to female gamete ratio could have a greater impact on fertilisation in vivo than in vitro.

Why is there the apparent need for LDH2 when LDH1 is already present in male gametocytes and microgametes? There are different reasons that could explain this. First, LDH1 is essential for blood stage parasite development^27,28^ and is presumably adapted to work optimally at 37^o^C. The large drop in temperature as the parasite transitions to the mosquito could reduce LDH1 activity to the point where it is unable to fully support the ATP production required for male gametogenesis. A second LDH activity with a lower temperature optimum would solve this issue. Second, fluctuations in ATP requirement may not be easily accommodated by a single LDH activity with fixed kinetic properties. A second LDH with more favourable enzyme kinetics could fulfil this need. For example, different affinities for pyruvate or lactate could allow LDH2 to operate at lower pyruvate or at higher lactate concentrations than LDH1, allowing ATP production during fast metabolic transitions or for fast-changing energy requirements (e.g. during cytokinesis). In this context it is worth noting that we were unable to functionally substitute LDH1 with LDH2 via allelic replacement (data not shown). This supports the notion that the two *Plasmodium* LDH proteins have distinct kinetic properties that are poorly interchangeable. This could reflect the low sequence homology between the substrate binding loops of *Plasmodium* LDH1 and LDH2 (Fig. 1, Supplementary Fig. S1).

Very recently, inhibitor studies in the human malaria parasite *P. falciparum* revealed a role for mitochondrial ATP production in male gametogenesis^23^. This is different from the situation in *P. berghei* where mitochondrial ATP production does not significantly contribute to male gamete formation^2^. This discrepancy could reflect differences between these two *Plasmodium* species. In both species, the *ldh1* gene is highly transcribed in intraerythrocytic ring and trophozoites stages, but not in gametocytes^29,30^ indicating that LDH1 activity is carried over into gametocytes from their precursor trophozoite stage. The much longer period required to complete gametocyte formation in *P. falciparum* (∼10 days) compared to *P. berghei* (∼1 day) could result in a decline in LDH1 activity that is compensated for by an increase in mitochondrial ATP production. In this scenario, LDH2 would likely be more important for microgamete motility and fertilisation in *P. falciparum*, but LDH2 knockout studies in this species are needed to test this hypothesis.

Our data reveal roles for both LDH2 and LDH1 in *P. berghei* male gametogenesis, identifying LDH activity as a potential transmission-blocking drug target. It has long been recognised that the distinctive insertion in the substrate binding loop of LDH1 (Fig. 1) sets it apart from its mammalian counterparts, making LDH1 an attractive therapeutic drug target^5,31–33^. Moreover, the three-dimensional structure of LDH1 shows that the inserted amino acid sequence forms a cleft next to the enzyme’s active site, which could accommodate inhibitor binding^5,31^ and we show here that similar structural features apply to LDH2 (Fig. 1, Supplementary Figs. S1, S2). Thus, our results warrant renewed efforts to find LDH-based antimalarial compounds.

## Materials and methods

### Ethics statement

As described previously^34^ all laboratory animal work was carried out in accordance with the United Kingdom Animals (Scientific Procedures) Act 1986 implementing European Directive 2010/63 for the protection of animals used for experimental purposes and was approved by the London School of Hygiene & Tropical Medicine Animal Welfare Ethical Review Body and United Kingdom Home Office. Experiments were typically conducted in 6–8 weeks old CD1 mice (Charles River Laboratories), specific pathogen free and maintained in individually ventilated cages, following ARRIVE guidelines. Animal welfare was assessed daily and upon reaching experimental or clinical endpoints animals were humanely euthanized by exposure to carbon dioxide gas in a rising concentration. Mice were infected with parasites suspended in phosphate buffered saline (PBS) by intraperitoneal injection, or by infected mosquito bite on anaesthetized animals (Xylazine, Ketamine). Intra-erythrocytic parasitemia was monitored regularly by microsampling blood from a superficial tail vein. Drugs were administered by intraperitoneal injection or where possible were supplied in drinking water. Parasitized blood was harvested by cardiac bleed under general anaesthesia without recovery.

### Parasite maintenance, culture and transmission

*Plasmodium berghei* ANKA clone 2.34 parasites were maintained as cryopreserved stabilates or by mechanical blood passage and regular mosquito transmission, as previously described^34^. Mosquito infection and transmission assays were as previously described using *Anopheles stephensi*^35,36^ and infected insects were maintained at 20 °C at approximately 70% relative humidity under a 12 h/12 h light/dark cycle. Ookinete cultures were set up overnight from gametocytemic blood as described^37^. For ookinete feeds, ookinetes were counted in neat cultures at 24 h post-set up, and the numbers between LDH2/GFP and LDH2-KO parasites were adjusted by diluting the culture with the higher ookinete concentration with an appropriate amount of a ‘naïve’ ookinete culture (set up using uninfected mouse blood). Cultures were then spun at low speed (800xg for 10 min at room temperature) and the cell pellets presented to mosquitoes in membrane feeders.

### Exflagellation assay

Whole blood (2 µL) was freshly collected from an infected mouse from a tail prick and was added to 100 µL exflagellation medium (ookinete medium supplemented with 10% foetal bovine serum and 1% heparin), mixed and kept at 20 °C. After 10 min, 10 µL was loaded into a haemocytometer and kept at 20 °C. After another 10 min, exflagellation events were counted and scored (number and size of observed exflagellation centres) using a light microscope with 40× objective.

### Macrogamete to ookinete conversion assay

Cells from 24 h-old ookinete cultures were labelled with anti-P28, fluorescein isothiocyanate-conjugated monoclonal antibody 12.1 (1:100 dilution) and examined by epifluorescence microscopy. Ookinete conversion was calculated as the percentage of P28-positive ookinetes to P28-positive spherical cells plus ookinetes.

### Generation and diagnostic PCR of transgenic parasite lines

To generate a DNA construct for fusing LDH2 to GFP, a 2.3 kb fragment corresponding to the *ldh2* gene plus 5’UTR (introns included) was PCR-amplified with primers LDH2-F (TTGGGCTGCAGTCGAGGTACCAACTCTCTAATACTTAAATGTGTACGTGC) and LDH2-R (ATGAGGGCCCCTAA*GCTAGC*ATTTGGCTTTGGTTCTTCCTC) and cloned into *Sal*I/*Hin*dIII digested plasmid pBS-EGFP-DHFR^34^ to give pBS-LDH2/GFP. A 850 bp fragment corresponding to the 3’UTR of the *ldh2* gene was then PCR-amplified with primers LDH2-3'UTR-F (ATATGCTAGAGCGGCCTGTATATGTATGATTGTGCGTGTG) and LDH2-3'UTR-R (CACCGCGGTGGCGGCCGCCACCACATGTAGA) (P2 in Fig. 2A) and cloned into *Not*I-digested pBS-LDH2/GFP to give pBS-LDH2/GFP/final. To generate a DNA construct for LDH2 knockout, pBS-LDH2/GFP/final was digested with *Nhe*I to excise entire ORF except for the first 15 codons, gel purified and recircularized with T4 DNA ligase to give pBS-LDH2-KO. This puts the first 15 codons of the LDH2 coding sequence in-frame with the GFP coding sequence. Plasmids pBS-LDH2/GFP/final and pBS-LDH2-KO were linearised with *Kpn*I and *Sac*II prior to transfection of purified schizonts to generate parasite lines LDH2/GFP and LDH2-KO, respectively, by double crossover homologous recombination (allelic replacement). Parasite transfection, pyrimethamine selection and limiting dilution cloning were performed as described^38,39^.

Primers LDH2-5prime-diag (GTATAATTTCTATAGGATATATTATATACATCCATTGAG) (P1 in Fig. 2A) and GFP-R (GTGCCCATTAACATCACC) (P3 in Fig. 2A) were used to PCR-amplify across the 5’ integration site with, which should amplify 2.4 kb in LDH2/GFP, and 1.1 kb in LDH2-KO. Primers LDH2-5prime-diag (P1) and LDH2-3'UTR-R (P2) were used to confirm absence of the wildtype *ldh2* allele, which should amplify a 3.1 kb fragment only in wildtype parasites.

To generate a DNA construct for replacing LDH1 with the GFP-tagged version of LDH2, approximately 0.9 kb of the *ldh1* 5’UTR was PCR amplified with primers LDH1-F3 (TAGGGCGAATTGGG*CTGCAG*GGAAAATTTAATACATGCCG) and LDH1-R (GTCGCTAGCACCTAGGATGCTAATTTTAGGATGTTTAACAGAAATCATTTTTTAAAAAGAAGCGGGGA) and cloned into *Pst*I/*Avr*II-digested pBS-LDH2/GFP to give pBS-LDH1/2/GFP. Approximately 0.8 kb of the *ldh1* 3’UTR was PCR amplified with primers LDH1-3'UTR-F (ATATGCTAGAGCGGCCGGATATTTTTAATTAAAATATGAGACTAATAAGAACAC) and LDH1-3'UTR-R (CACCGCGGTGGCGGCCATGAGCGTATGAATGTTACATTAGAAC) and cloned into *Not*I-digested pBS-LDH1/2/GFP to give pBS-LDH1/2/GFP/final. This plasmid was linearized with *Pst*I and *Sac*II prior to transfection. Primers LDH1-5prime-diag (CTTCTTATTAAAATAAGAATCTATAAATATAATAATAACTTTCC) and GFP-R were used to assess integration across the 5’-integration site, which should amplify approximately 2.6 kb.

### Polymerase chain reaction

PCR was carried out with Advantage 2 DNA Polymerase Mix (Takara) according to manufacturer’s instructions, typically for 25 cycles of combined denaturation (30 s, 94 °C), annealing (30 s, 50 °C) and extension (62 °C, 1 min per kb), following an initial 2 min round of denaturation at 94 °C.

### Bioinformatics

Multiple sequence alignment was carried out with multiple sequence comparison by log-expectation (MUSCLE)^40^. Pairwise structure alignment was carried out using the jCE-CP method^41^ in the RCSB protein data bank (www.rcsb.org). AlphaFold structures of LDH2 were identified by searching the AlphaFold Protein Structure Database (https://alphafold.ebi.ac.uk/) with the amino acid sequence. Phylogeny of LDH/MDH proteins was obtained by Neighbor Joining (Bootstrap *n* = 1000, Poisson correction and gaps distributed proportionally) using MacVector version 18.5.1 software.

### Live fluorescence imaging

Live parasite samples were assessed, and images captured, on a Zeiss LSM880 laser scanning confocal microscope using an 100x oil objective and ZEN software (Zeiss), as described^34^. At least two clonal populations of transgenic parasites were compared to ensure results were consistent and representative.

### Immunofluorescence

Parasitised blood of high gametocytemia was diluted 10-fold in exflagellation medium. Cells were collected, thinly spotted onto glass microscope slides and allowed to air dry, followed by 5 min fixation in 100% methanol. Slides were blocked for 1 h in PBS supplemented with 1% BSA, 10% normal goat serum and 0.1% Tween, followed by incubation in anti-alpha tubulin monoclonal antibody (clone DM1A, Sigma) diluted 500-fold in PBS supplemented with 1% BSA and 0.1% Tween for 1 h at 37 °C. After two 15 min washes cell were incubated in goat anti-mouse IgG (H + L) conjugated to Alexa Fluor 488, diluted 1000-fold in PBS supplemented with 1% BSA and 0.1% Tween for 1 h at room temperature. After a further two washes, cells were mounted and examined by fluorescence microscopy.

## Electronic supplementary material

Below is the link to the electronic supplementary material.

Supplementary Material 1

Supplementary Material 2

Supplementary Material 3

Supplementary Material 4

## Data Availability

Data generated or analysed during this study are included in this published article and its Supplementary Information files.
