# Supplementary material for: A paternal lactate dehydrogenase critically enhances male gametogenesis and malaria transmission
Source: Sci Rep. 2025 Jul 2;15:23283. doi: 10.1038/s41598-025-05832-1 (PMC12223287; doi:10.1038/s41598-025-05832-1)
Supplement: Supplementary file 1 — Supplementary Material 1 [file 41598_2025_5832_MOESM1_ESM.pdf]

Supplementary Fig. S1. ***Plasmodium* encodes a second lactate dehydrogenase (LDH).** (**A**) Multiple sequence alignment of LDH and malate dehydrogenase (MDH) from *Plasmodium*, *Toxoplasma* and human. The substrate binding loops are indicated in red font. Canonical catalytic residues are shown in bold. PfLDH1, *P. falciparum* LDH1 (PF3D7_1324900); PvLDH1, *P. vivax* LDH1 (PVP01_1229700); PkLDH1, *P. knowlesi* LDH1 (PKNH_1203900); PbLDH1, *P. berghei* LDH1 (PBANKA_1340100); TgLDH1, *T. gondii* LDH1 (TGME49_232350); TgLDH2, *T. gondii* LDH2 (TGME49_291040); PfLDH2, *P. falciparum* LDH2 (PF3D7_1325200); PvLDH2, *P. vivax* LDH2 (PVP01_1229400); PkLDH2, *P. knowlesi* LDH2 (PKNH_1203600); PbLDH2, *P. berghei* LDH2 (PBANKA_1340400); PfMDH, *P. falciparum* MDH (PF3D&_0618500); PvMDH, *P. vivax* MDH (PVPo1_1131000); PkMDH, *P. knowlesi* MDH (PKNH_1131900); PbMDH, *P. berghei* MDH (PBANKA_1117700); TgMDH, *T. gondii* MDH (TGME49_318430); HsLDH, *Homo sapiens* lactate dehydrogenase A (KAI4070372).

```
PfLDH2     1               MTSVKHPKISVLGAGDIGCALAHMICEKNLGD-VVLHDFRKDLPKG  45
PvLDH2     1               MTSVKHPKISVLGAGDIGCTLAHMICEKNLGD-VVLHDFRKDLPKG  45
PkLDH2     1               MTSVKHPKISVLGAGDIGCTLAHMICEKNLGD-VVLHDFTKDLPKG  45
PbLDH2     1               MISVKHPKISILGASDIGCTLAHMICEKNLGD-VVLHDFRKDLSKG  45
PfLDH1     1                 MAPKAKIVLVGSGMIGGVMATLIVQKNLGD-VVLFDIVKNMPHG  43
PvLDH1     1                 MTPKPKIVLVGSGMIGGVMATLIVQKNLGD-VVMFDVVKNMPQG  43
PkLDH1     1                 MAPKPKIVLVGSGMIGGVMATLIVQKNLGD-VVMFDVVKNMPQG  43
PbLDH1     1                 MAPKAKIVLVGSGMIGGVMATLIVQKNLGD-VVMFDIVKNMPHG  43
TgLDH1     1            MAPALVQRRKKVAMIGSGMIGGTMGYLCALRELAD-VVLYDVVKGMPEG  48
TgLDH2     1             MTGTVSRRKKIAMIGSGMIGGTMGYLCVLRELAD-VVLFDVVTGMPEG  47
TgMDH      1                  MSRRKIGLIGGGNIGATLALLSAVKELGD-VVMFDVVQDLPQG  42
PfMDH      1                    MTKIALIGSGQIGAIVGELCLLENLGD-LILYDVVPGIPQG  40
PvMDH      1                    MPKISMVGSGQIGAIVGQLILMENIGD-IVLYDVMQGVPQG  40
PkMDH      1                    MTKISMIGSGQIGTIVGQLILMENIGD-IVLYDVMQGVPQG  40
PbMDH      1                    MPKISLIGSGQIGAIVGQLCLSENIGD-IVLYDVVNGIPQG  40
HsLDH      1 MATLKDQLIYNLLKEEQTPQNKITVVGVGAVGMACAISILMKDLADELALVDVIEDKLKG  60
                                  *. ..*   .*           . * . . *       *

PfLDH2    46 RALDILHTRPLNRSRINILGTNEIT-DIKDSLVVVVTIEVSEREFAEFDEEDLEKQVYTS 104
PvLDH2    46 RALDILHTRPLNRSRINILGTNEIT-DIKDSLVVVVTIEVSEREFAEFDEEDIERQVYTS 104
PkLDH2    46 RALDILHTRPLNRSRINILGTNEIT-DIKDSLVVVVTIEVSEREFAEFDEEDIERQVYTS 104
PbLDH2    46 RALDILHTRPINRSKINILGTSDIT-DIKDSLVVVVTIEVSEREFAEFDEEDIEKQVYTS 104
PfLDH1    44 KALDTSHTNVMAYSNCKVSGSNTYD-DLAGADVVIVTAGFTKAP-GKSDKEWNRDDLLPL 101
PvLDH1    44 KALDTSHSNVMAYSNCKVTGSNSYD-DLKGADVVIVTAGFTKAP-GKSDKEWNRDDLLPL 101
PkLDH1    44 KALDTSHSNVMAYSNCKVTGSNSYE-DLEGADVVIVTAGFTKAP-GKSDKEWNRDDLLPL 101
PbLDH1    44 KALDTSHTNVMAYSNCKVSGSNTYD-DLKDADVVIVTAGFTKAP-GKSDKEWNRDDLLPL 101
TgLDH1    49 KALDLSHVTSVVDTNVSVRAEYSYEAALTGADCVIVTAGLTKVP-GKPDSEWSRNDLLPF 107
TgLDH2    48 KALDDSQATSIADTNVSVTSANQYE-KIAGSDVVIITAGLTKVP-GKSDKEWSRNDLLPF 105
TgMDH     43 KCLDLYQLTPISGVDVRFEGSNDYS-VLKDADVIIVTAGVPRKP-GM-----SRDDLLAI  95
PfMDH     41 KALDLKHFSTILGVNRNILGTNQIE-DIKDADIIVITAGVQRKE-GM-----TREDLIGV  93
PvMDH     41 KSLDLKHFSTIVGVNKNILGTNNVQ-DIKDSDVIVITAGVQRKE-GM-----TREDLIGI  93
PkMDH     41 KSLDLKHFSTIVGVNKKIVGTNNVE-DIKDSDVIVITAGVQRKE-GM-----TREDLIGI  93
PbMDH     41 KSLDLKHYSTIIGVNRKIIGTNNIK-DITDSDVIVITAGVQRKE-GM-----SREDLIGI  93
HsLDH     61 EMMDLQHGSLFLRTPKIVSG-KDYN-VTANSKLVIITAGARQQE-GE-----SRLNLVQR 112
               .*  .                       .  ...*                   .

PfLDH2   105 NVKLLKEVAKSLKKHCPQAFVVVTTSPVDCMAKVLQEHANIPPHKICGMAGVLHSARLRH 164
PvLDH2   105 NVKLLKEVSKSIKKHCPQAFVVVTTNPVDCMAKVLQENANIPSHKICGMAGVLHSARLRH 164
PkLDH2   105 NVKLLKEVSKSIKKHCPQAFVVVTTNPVDCMAKVLQVHGNIPSHKICGMAGVLHSARLRH 164
PbLDH2   105 NVKLLKDVSKAIKKHCPHAFVVVTTNPVDCMAKVLQDYANIPSHKICGMAGVLHSARLRH 164
PfLDH1   102 NNKIMIEIGGHIKKNCPNAFIIVVTNPVDVMVQLLHQHSGVPKNKIIGLGGVLDTSRLKY 161
PvLDH1   102 NNKIMIEIGGHIKNLCPNAFIIVVTNPVDVMVQLLFEHSGVPKNKIIGLGGVLDTSRLKY 161
PkLDH1   102 NNKIMIEIGGHIKKLCPNAFIIVVTNPVDVMVQLLFEHSGVPKNKIIGLGGVLDTSRLKY 161
PbLDH1   102 NNKIMIEIGGHIKNNCPNAFIIVVTNPVDVMVQLLHQHSGVPKNKIVGLGGVLDTSRLKY 161
TgLDH1   108 NSKIIREIGQNIKKYCPKTFIIVVTNPLDCMVKVMCEASGVPTNMICGMACMLDSGRFRR 167
TgLDH2   106 NAKIIREVAQGVKKYCPLAFVIVVTNPLDCMVKCFHEASGLPKNMVCGMANVLDSARFRR 165
TgMDH     96 NAKIMGQVGEAIKQYCPNAFVICITNPLDVMVYILREKCGLPPHKVCGMAGVLDSARLRT 155
PfMDH     94 NGKIMKSVAESVKLHCSKAFVICVSNPLDIMVNVFHKFSNLPHEKICGMAGILDTSRYCS 153
PvMDH     94 NGKIMKSVAESVKVHSPNAFVICVSNPLDIMVNVFQKYSGLPHEQICGMAGILDTSRFRT 153
PkMDH     94 NGKIMKSVAESVKLYSPNAFVICVSNPLDIMVNVFHKYSGLPYEKICGMAGILDTSRFRS 153
PbMDH     94 NGKIIKSVAESVKQYAPNAFVICVTNPLDVMVNVFHKYSNLPYEKICGMAGILDTSRFRY 153
HsLDH    113 NVNIFKFIIPNVVKYSPNCKLLIVSNPVDILTYVAWKISGFPKNRVIGSGCNLDSARFRY 172
             *  .   .   .   .  . ..  . *.* .        . *   . *    * . *
```

```
PfLDH2   165 NLAEKLRVNPGDVQGFVIGAHGDKMVPLPRYCCVNGIPLSDFTKK--GAITEKEINQIVE 222
PvLDH2   165 NLAEKLRVNPGDVQGFVIGAHGDKMVPLPRYCCVNGIPLHDFTKK--GAITEKEISQIVE 222
PkLDH2   165 NLAEKLRVNPGDVQGFVIGAHGDKMVPLPRYCCVNGIPLHDFTKK--GAITEKEISQIVE 222
PbLDH2   165 NLAEKLRVNPGDVQGFVIGAHGDKMVPLPRYCCVNGIPLCDFTKK--GAITEKEISKIVE 222
PfLDH1   162 YISQKLNVCPRDVNAHIVGAHGNKMVLLKRYITVGGIPLQEFINN--KLISDAELEAIFD 219
PvLDH1   162 YISQKLNVCPRDVNALIVGAHGNKMVLLKRYITVGGIPLQEFINN--KKITDEEVEGIFD 219
PkLDH1   162 YLSQKLNVCPRDVNALIVGAHGNKMVLLKRYITVGGIPLQEFINN--KKITDEEVEAIFD 219
PbLDH1   162 YISQKLNVCPRDVNAHIVGAHGNKMVLLKRYITVGGIPLQEFINN--KKITDQELDAIFD 219
TgLDH1   168 YVADALSVSPRDVQATVIGTHGDCMVPLVRYITVNGYPIQKFIKD--GVVTEKQLEEIAE 225
TgLDH2   166 FIADQLEISPRDIQATVIGTHGDHMLPLARYVTVNGFPLREFIKK--GKMTEAKLAEIVE 223
TgMDH    156 FLSERLNVSVDDIHALVMGGHGDTMVPLPRFTTVGGIPLPELVKM--GMISQQEVDDIVQ 213
PfMDH    154 LIADKLKVSAEDVNAVILGGHGDLMVPLQRYTSVNGVPLSEFVKK--NMISQNEIQEIIQ 211
PvMDH    154 LLAEKLKVAPQNVSQVLLGGHGDLMVPLERYCSISGIPLSEFVKK--NLISKEEINEIVK 211
PkMDH    154 LLGEKLNVAPENVNLVLLGGHGDLMVPLKRYCSVSGIPLSDFIEK--KLITNEEINDIIK 211
PbMDH    154 LLSEKLNVSPENINAIILGGHGDLMMPLPRYCSISGIPLLDYIKN--HDMSEKDISDIIE 211
HsLDH    173 LMGERLGVHPLSCHGWVLGEHGDSSVPVWSGMNVAGVSLKTLHPDLGTDKDKEQWKEVHK 232
              .   * .        ..* **   . .     . *  .                  .

PfLDH2   223 KTRNTGFELLELLPEGSVCFAPSLAIVEIIEAYLKDLKRVLVCSVLLNGHYGHK-GVFAG 281
PvLDH2   223 KTRNTGLELLELLPEGSVCFAPSLAIVEIIEAYLKDLKRVLVCSVPLNGQYGHK-GVFAG 281
PkLDH2   223 KTRNTGLELLDLLPEGSVCFAPSLAIVEIIEAYLKDLKRVLVCSVPLNGQYGHK-GVFAG 281
PbLDH2   223 KTKNTSLELLDLMPEGSVCFAPSSAIVEIIEAYLKDLKRVLVCSIHLNGQYGHK-GVFAG 281
PfLDH1   220 RTVNTALEIVNLHA--SPYVAPAAAIIEMAESYLKDLKKVLICSTLLEGQYGHS-DIFGG 276
PvLDH1   220 RTVNTALEIVNLLA--SPYVAPAAAIIEMAESYLKDIKKVLVCSTLLEGQYGHS-NIFGG 276
PkLDH1   220 RTVNTALEIVNLLA--SPYVAPAAAIIEMAESYLKDIKKVLVCSTLLEGQYGHK-NIFGG 276
PbLDH1   220 RTINTALEIVNLHA--SPYVAPAAAIIEMAESYIRDLRKVLICSTLLEGQYGHK-DIFAG 276
TgLDH1   226 HTKVSGGEIVRFLGQGSAYYAPAASAVAMATSFLNDEKRVIPCSVYCNGEYGLK-DMFIG 284
TgLDH2   224 RTKKAGGEIVRLLGQGSAYYAPALSAITMAQAFLKDEKRVLPCSVYCQGEYGLH-DMFIG 282
TgMDH    214 RTRNGGGEIVSLLKTGSAFFAPAAAGVLMAEAYLKDRKRVLPCAAYLNGEYGVK-DMYVG 272
PfMDH    212 KTRNMGAEIIKLAKA-SAAFAPAAAITKMIKSYLYNENNLFTCAVYLNGHYNCS-NLFVG 269
PvMDH    212 QTRDMGSQIIKLAKS-SATFAPAAAIVKMIKSFLFNESQLYTCAVYLNGLYNCS-DLYVG 269
PkMDH    212 KTRDMGAEIIKLSKS-SATFSPAAAIVKMIKSYLYNESQLYTCAVYLNGLYNCS-NLYVG 269
PbMDH    212 KTRNMGGEIIKLAKS-SAAFAPAASIIKMIKSYLHDQNQLFTCAVYLSGLYNCK-DLYAG 269
HsLDH    233 QVVESAYEVIKLKG--YTSWAIGLSVADLAESIMKNLRRVHPVSTMIKGLYGIKDDVFLS 290
             .      ...          .   .   .  . .     .   .    * *.    ..

PfLDH2   282 IPVVIGGKGIEKIIELDLNTQEKELFDDSLKHISYLFDNYKHETVVDDENKPN 334
PvLDH2   282 IPVVIGGKGIEKIIELDLNAQEKELFDDSLKHISYLFENYKHEAVVEEEAKPN 334
PkLDH2   282 VPVVIGGKGIEKIIELDLNAQEKELFDDSLKHISYLFENYKHEAVVDEEAKPN 334
PbLDH2   282 VPVVIGGKGIEKVIELDLNTQEKELFDDSLKHISYLFDNYKHESAIEEEPKPN 334
PfLDH1   277 TPVVLGANGVEQVIELQLNSEEKAKFDEAIAE----TKRMKALA          316
PvLDH1   277 TPLVIGGTGVEQVIELQLNAEEKTKFDEAVAE----TKRMKALI          316
PkLDH1   277 TPLVIGGTGVEQVIELQLTAEEKAKFDEAVAE----TKRMKALI          316
PbLDH1   277 TPLVIGGNGVEQVIELQLNADEKKKFDEAVAE----TSRMKALI          316
TgLDH1   285 LPAVIGGAGIERVIELELNEEEKKQFQKSVDDVMALNKAVAALQA         329
TgLDH2   283 LPAVIGGGGIEQVIELELTHEEQECFRKSVDDVVELNKSLAALG          326
TgMDH    273 VPCVIGAGGVEKIVELDLTPEEKKMFERSVESVKTLLAAAPKSA          316
PfMDH    270 STAKINNKGAHPV-EFPLTKEEQDLYTESIASVQSNTQKAFDLIK         313
PvMDH    270 TTAIINSSGAKPI-EFALTQEEQQLYDKSIAFVREHTQKAFALIN         313
PkMDH    270 STAIINSSGAKAI-EFALTKEEQELYDKSISFVHEHTQKAFALIN         313
PbMDH    270 STAIINKTGAHPI-EFILTEEEQACYQKSINNIRENTQKALNVID         313
HsLDH    291 VPCILGQNGISDLVKVTLTSEEEARLKKSADTLWGIQKELQF            332
                 ..  *   .    *.  *      .
```
