# Supplementary material for: A paternal lactate dehydrogenase critically enhances male gametogenesis and malaria transmission
Source: Sci Rep. 2025 Jul 2;15:23283. doi: 10.1038/s41598-025-05832-1 (PMC12223287; doi:10.1038/s41598-025-05832-1)

Supplementary Fig. S2. Pairwise structure alignment of *Plasmodium berghei* LDH1 (PDB: 1OC4, maroon) and the AlphaFold-predicted structure of *P. berghei* LDH2 (AF: A0A5009AR16, blue). Amino acid identity is 42% across 308 aligned residues, root mean square deviation is 1.25. Substrate binding loops are highlighted in red (LDH1) and yellow (LDH2).

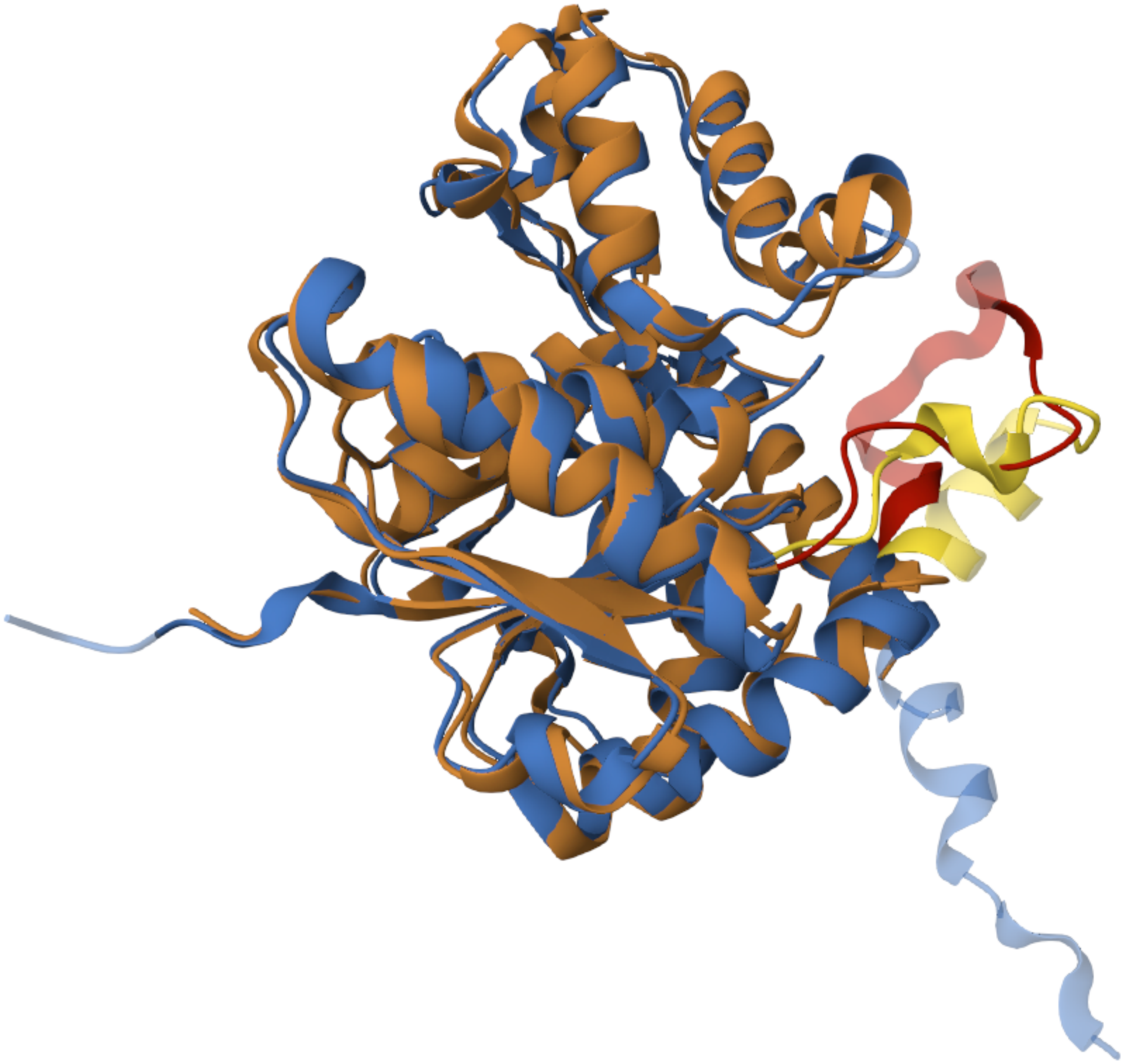

Supplement: Supplementary file 2 — Supplementary Material 2 [file 41598_2025_5832_MOESM2_ESM.pdf]
