# Supplementary material for: A paternal lactate dehydrogenase critically enhances male gametogenesis and malaria transmission
Source: Sci Rep. 2025 Jul 2;15:23283. doi: 10.1038/s41598-025-05832-1 (PMC12223287; doi:10.1038/s41598-025-05832-1)

Supplementary Fig. S3. Uncropped DNA agarose gels corresponding to Figure 2B. White boxes mark cropped regions. DNA size markers are shown on the left-hand side.

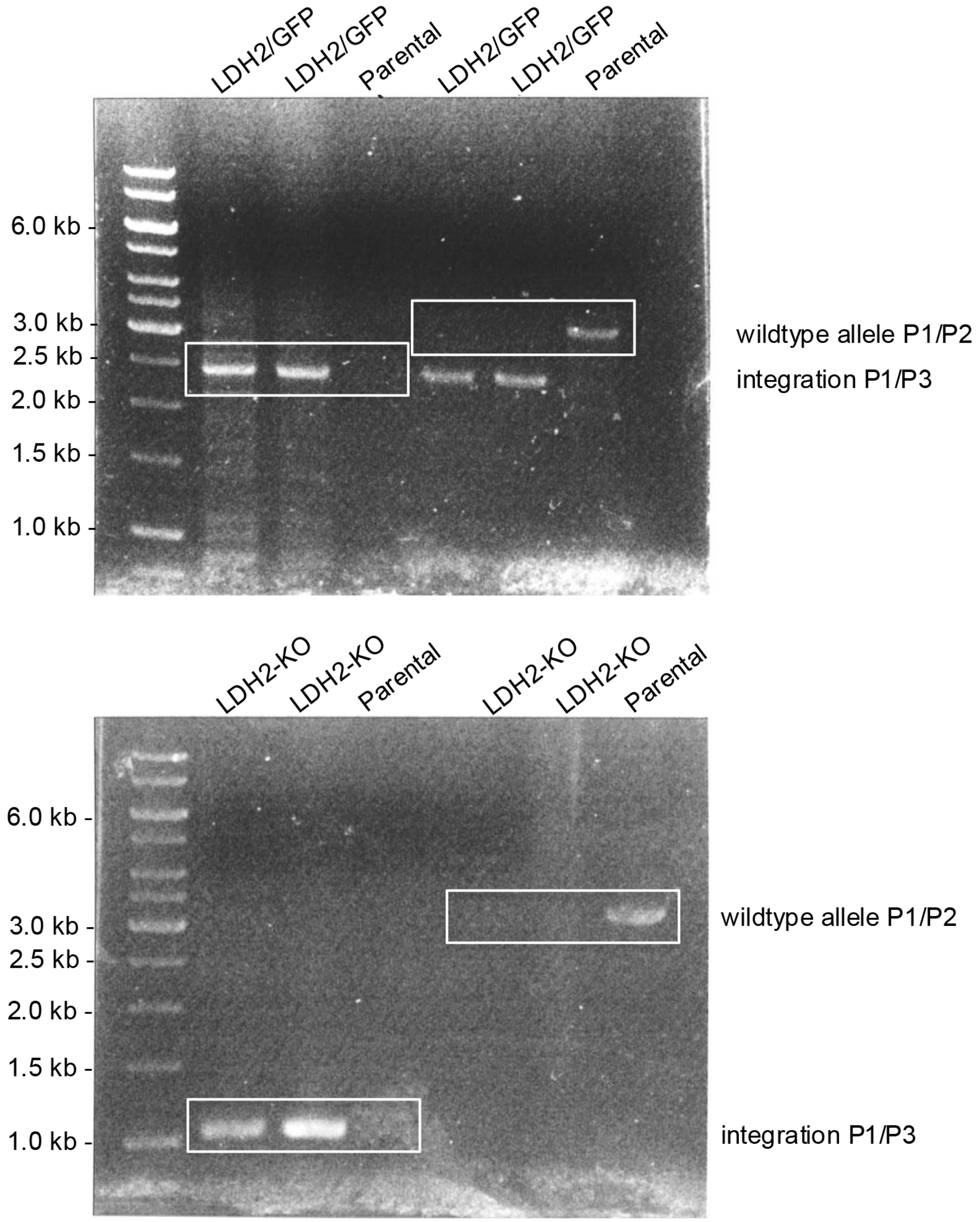

Supplement: Supplementary file 3 — Supplementary Material 3 [file 41598_2025_5832_MOESM3_ESM.pdf]
