# Supplementary material for: A paternal lactate dehydrogenase critically enhances male gametogenesis and malaria transmission
Source: Sci Rep. 2025 Jul 2;15:23283. doi: 10.1038/s41598-025-05832-1 (PMC12223287; doi:10.1038/s41598-025-05832-1)

Supplementary Fig. S4. LDH2/GFP and LDH2-KO gametocytes subjected to immunofluorescence imaging with anti-tubulin antibodies to visualize the flagella. Cells were fixed 20 min after gametocyte activation.

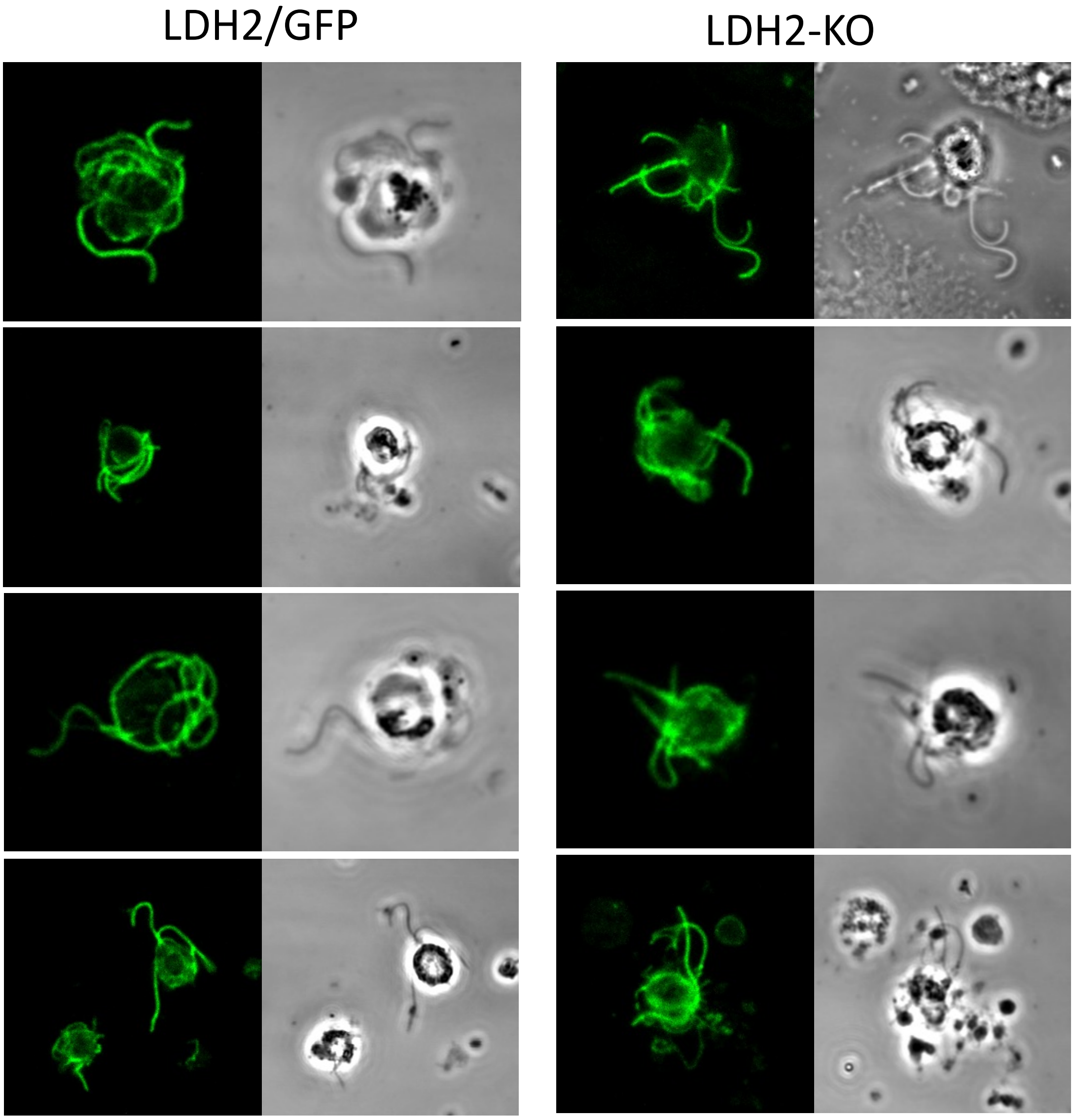

Supplement: Supplementary file 4 — Supplementary Material 4 [file 41598_2025_5832_MOESM4_ESM.pdf]
